# Intratesticular Angiolipoma: A Rare Case of Adipose Tissue Presence in the Testis

**DOI:** 10.1155/2019/7606530

**Published:** 2019-12-23

**Authors:** V. Kalyvas, C. Gkekas, D. Papadopoulos, A. Malioris, S. Milias, M. Papathanasiou, N. Kalinderis, K. Moysidis, K. Hatzimouratidis

**Affiliations:** ^1^Department of Urology, 424 General Military Hospital of Thessaloniki, Greece; ^2^2nd Department of Urology, Aristotle University of Thessaloniki, Greece; ^3^Department of Pathology, 424 General Military Hospital of Thessaloniki, Greece

## Abstract

**Introduction:**

Solid, fat-containing tumors of the testes are extremely rare with only a few cases having been reported so far, contrary to the more frequent occurrence of paratesticular lipomatosis. Testicular angiomyolipomas and gonadal involvement in Cowden's disease, although infrequently occurring, are known examples of fat-bearing testicular lesions. Hereby, we present an extremely rare case of intratesticular angiolipoma. Angiolipomas are benign tumors of the subcutaneous tissue commonly occurring in the trunk and the extremities. Histologically, they are characterized by ample vascularity and an excess of mature adipocytes. Definitive diagnosis is established by biopsy of the lesion.

**Presentation of the Case:**

A 35-year-old patient presented to our andrology outpatient clinic for fertility assessment. Physical examination of external genitalia revealed no significant pathology. Testicular ultrasound however depicted an isoechoic lesion on the upper pole of the right testis measuring 1.8 cm × 0.8 cm × 1 cm and exhibiting intense arterial flow. After sonographic and MRI investigation, the patient was referred for semen analysis and cryopreservation. Subsequently, the patient underwent testicular biopsy (frozen section biopsy) and right partial orchiectomy. Final histology reported a noninfiltrating testicular angiolipoma. No recurrences have been observed in the follow-up period.

**Discussion:**

Angiolipomas, which mainly occur in the trunk and extremities, are classified as infiltrating and noninfiltrating. The diagnosis is based on both clinical and histologic criteria, and the main method of treatment for both types is by surgical excision. The infiltrating type exhibits higher recurrence rates.

**Conclusion:**

Angiolipomas commonly occur in the subcutaneous tissue and have been invariably treated by radical excision. Hereby, we report the first case of an intratesticular, noninfiltrating type angiolipoma which was treated by partial orchiectomy.

## 1. Introduction

Angiolipomas are benign tumors, commonly occurring in the subcutaneous tissue. They are either encapsulated or nonencapsulated fatty tumors and are distinguished from other lipomas by the excessive degree of vascular proliferation and abundance of mature adipocytes. They more often occur in the trunk and extremities, rarely in the maxillofacial area [1] and are usually diagnosed histologically [2]. We report the first case of an intratesticular angiolipoma.

## 2. Case Presentation

A 35-year-old patient presented to our andrology outpatient clinic for fertility assessment. Physical examination of the external genitalia was insignificant. There was neither history of testicular trauma nor any sexually transmitted disease in his medical record.

**Figure 1:**
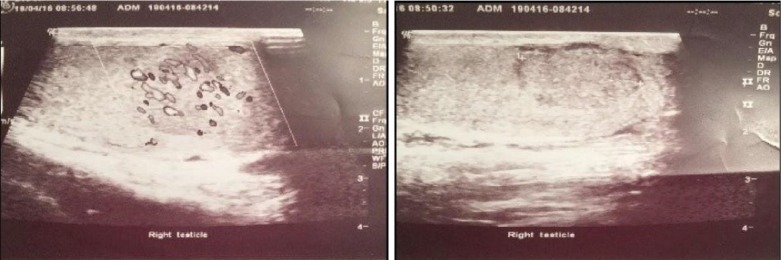
Scrotal ultrasound: right testicle.

Testicular ultrasound revealed a solitary isoechoic, highly vascularized lesion of the upper pole of the right testis measuring 1.8 cm × 0.8 cm × 1 cm distinctly separated from the adjacent healthy parenchyma by a well-defined hypoechoic capsule (Figure 1). No additional testicular or epididymal pathology was identified on both sides.

Hormonal testing (FSH, LH, Testosterone, *β*-hCG, and aFP) and semen analysis fell within the normal range (Tables 1 and 2).

CT scan of the thorax, abdomen, and pelvis revealed no retroperitoneal lymphadenopathy or other abnormalities.

MRI of the scrotum demonstrated a well-defined encapsulated mass, located in the upper pole of the right testis measuring 1.9 cm × 0.9 cm × 1.2 cm.

**Figure 2:**
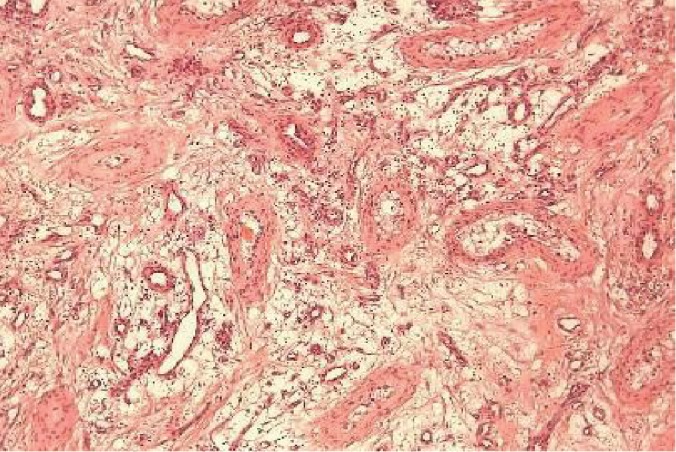
Testicular tumor composed of mature adipose tissue and small vessels: seminiferous tubules with thickened basement membrane and atrophic are also observed (H&E stain ×10).

**Figure 3:**
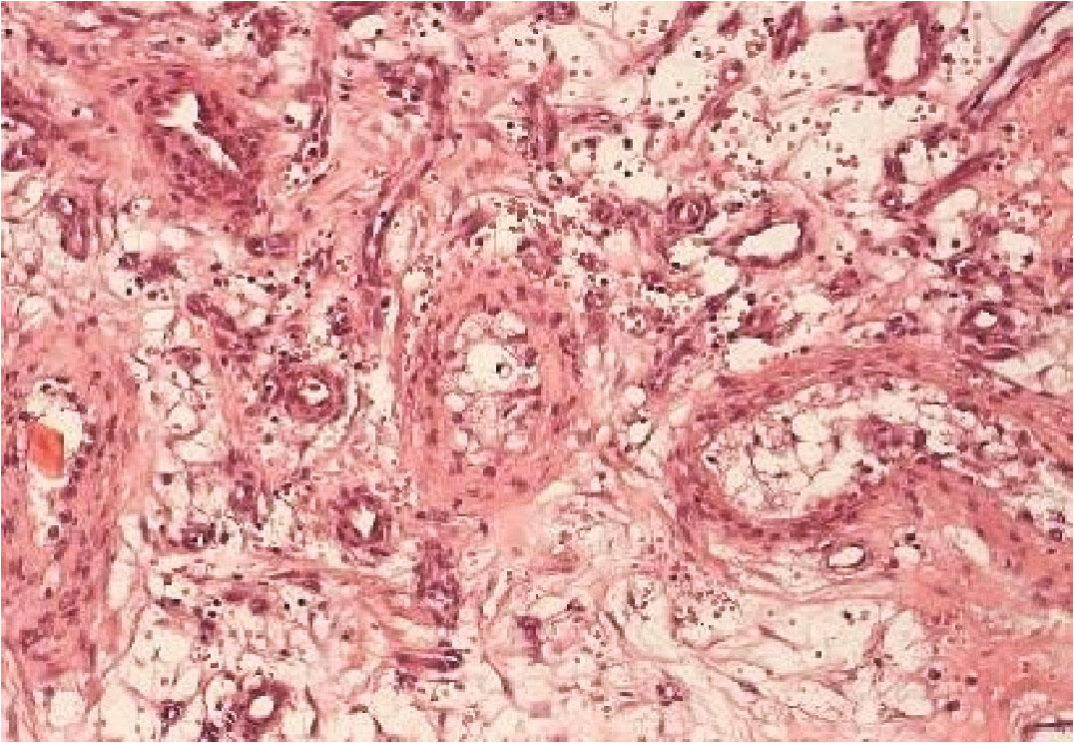
Same area under higher magnification (H&E stain ×20).

**Figure 4:**
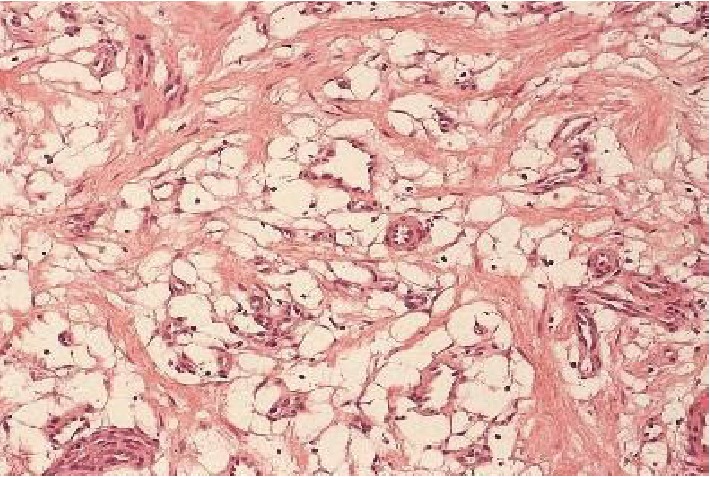
Area with numerous adipocytes and small vessels, some of them including erythrocytes in their lumen (H&E stain ×20).

**Figure 5:**
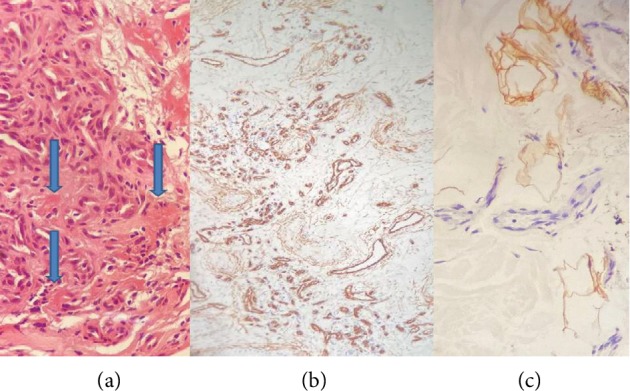
(a) Microthrombi in capillaries (blue arrows) (H&E stain ×20). (b) Endothelial cells of the vascular capillaries are positive (CD34 immunohistochemistry ×10). (c) Adipocytes are positive (S100 Protein Immunohistochemistry ×40).

After semen cryopreservation, the patient underwent surgical exploration. Intraoperative frozen section analysis reported a nonmalignant lesion, possibly an angiolipoma. A right partial orchiectomy was subsequently performed. The final histology reported a 1.9 cm × 0.9 cm × 1.2 cm lesion composed of adipocytes and small vessels with fibrous microthrombi, identified as noninfiltrating type angiolipoma. The tumor was circumscribed and partially encapsulated. Capillaries and mature adipocytes were observed. Few vascular channels contained microthrombi. Around the periphery of the tumor, the capillaries and the adipocytes entrapped the seminiferous tubules. This finding, which is also observed in testicular hemangiomas, perhaps predicts a more aggressive behavior of the tumor with higher possibility of recurrence in cases of partial orchiectomy (Figures 2–5).

Follow-up visits included physical examination, hormonal assessment, and scrotal ultrasound every three months for the first two years, every six months for the next two years, and subsequently on a yearly basis. The patient has currently been free of recurrence.

## 3. Discussion

Angiolipomas consist a variant of lipomas which was first described by Bowen in 1912 [1]. In 1960, Howard [3] classified angiolipomas as a new entity, because of their distinct clinical and pathological differences with lipomas. The presence of adipocyte cells in the testis has previously been described in angiomyolipomas (AML), which are mostly benign mesenchymal tumors composed of fat, smooth muscle cells, and tortuous, thick-walled blood vessels [4, 5]. Likewise, in testicular lipomatosis or Cowden's disease, nested fat cells have been identified in the testicular parenchyma [6]. Additionally, fat cells have been reported in Leydig cell tumors, coupled with atypical features, such as calcification, ossification, and spindle cell proliferation [7].

Based on cytogenetic analysis, the karyotype of angiolipomas differs from that of other benign lipomatous tumors [8] which suggests that the hamartomatous origin of angiolipomas may be different from the rest of the lipomas. They commonly develop in the trunk and extremities and rarely in the gastrointestinal tract [1]. They are usually encountered as multiple, small tumors (less than 2 cm in diameter each), which produce no symptoms [9]. There is no reference in the existing literature of testicular angiolipomas.

Angiolipomas can easily be distinguished from other lipomas from their excess of vascular elements [10]. There are various factors that are related to the etiology of angiolipomas such as a history of trauma, vascular transformation of a lipoma, and hormonal imbalances [3].

J. J. Lin and F. Lin classified angiolipomas into infiltrating and noninfiltrating types [8]. The noninfiltrating type which is the most common occurs in younger individuals and presents as a painless subcutaneous nodule [8, 10]. Histologically, it is encapsulated and consists a mixture of mature adipocytes interspersed between thin-walled vessels [11]. The infiltrating angiolipoma which rarely has a capsule occurs in older patients, invades into the adjacent structures, and is more common in the head and neck region [10]. The diagnosis is based on both clinical and histological criteria (Table 3).

The main treatment for both types is by complete excision. Noninfiltrating angiolipomas have no tendency to recur after surgical removal; therefore, a simple excision of the lesion will probably be curative. In the case of infiltrating angiolipomas, the recurrence rate is higher and a wide surgical excision is imperative [8, 12, 13].

## 4. Conclusion

Angiolipomas are benign tumors, commonly occurring in the subcutaneous tissue. This is the first recorded case of an intratesticular, noninfiltrating type angiolipoma which was treated by organ-sparing surgery. Two years after the procedure, there were no recurrences. The rarity of the specific entity and the lack of published experience impede the construction of an evidence-based treatment algorithm. Every case report adds to the growing body of evidence.

## Figures and Tables

**Table 1:** Hormonal testing.

FSH	2.17 mIU/ml
LH	3.7 mIU/ml
Testosterone	3.1 ng/ml
*β*-hCG	<1.2 mIU/ml
aFP	3.7 ng/ml

**Table 2:** Semen analysis.

Ejaculate volume (ml)	2.1
pH	7.3
Total sperm number (10/ejaculate)	29.4
Sperm concentration (10/ml)	14
Total motility (PR+NP)	39
Progressive motility (PR, %)	32
Vitality (live spermatozoa, %)	59
Sperm morphology (normal forms, %)	4

PR = progressive; NP = nonprogressive.

**Table 3:** Histologic guidelines for diagnosis of noninfiltrating angiolipoma [1].

Evidence of 50% mature adipocytes in the tumor
Interspersed angiomatous proliferation in the tumor
Well encapsulated
Fibrinous microthrombi
Absence of other mesenchymal elements

## References

[1] Arenaz Búa J., Luáces R., Lorenzo Franco F. (2010). Angiolipoma in head and neck: report of two cases and review of the literature. *International Journal of Oral and Maxillofacial Surgery*.

[2] Wang L., Chen P., Zong L., Wang G. Y., Wang H. (2013). Colon angiolipoma with intussusception: a case report and literature review. *World Journal of Surgical Oncology*.

[3] Pattipati S., Kumar M. N., Ramadevi B. P. K. (2013). Palatal lipoma: a case report. *Journal of Clinical and Diagnostic Research*.

[4] Nelson C. P., Sanda M. G. (2002). Contemporary diagnosis and management of renal angiomyolipoma. *The Journal of Urology*.

[5] Cibas E., Goss G., Kulke M., Demetri G., Fletcher C. (2001). Malignant epithelioid angiomyolipoma (`sarcoma ex angiomyolipoma') of the kidney: a case report and review of the literature. *The American Journal of Surgical Pathology*.

[6] Woodhouse J. B., Delahunt B., English S. F., Fraser H. H., Ferguson M. M. (2005). Testicular lipomatosis in Cowden’s syndrome. *Modern Pathology*.

[7] Ulbright T. M., Srigley J. R., Hatzianastassiou D. K., Young R. H. (2002). Leydig cell tumors of the testis with unusual features. Adipose differentiation, calcification with ossification, and spindle-shaped tumor cells. *The American Journal of Surgical Pathology*.

[8] Lin J. J., Lin F. (1974). Two entities in angiolipoma(*A study of 459 cases of lipoma with review of literature on infiltrating angiolipoma*). *Cancer*.

[9] Davis G. B., Stoelinga P. J. W., Tideman H., Bronkhorst F. (1976). Angiolipoma of the hard palate: a case report and review of the literature. *Journal of Maxillofacial Surgery*.

[10] Flaggert J. J., Heldt L. V., Keaton W. M. (1986). Angiolipoma of the palate: Report of a case. *Oral Surgery, Oral Medicine, Oral Pathology*.

[11] Reilly J. S., Kelly D. R., Royal S. A. (1988). Angiolipoma of the parotid: case report and review. *The Laryngoscope*.

[12] Dalambiras S., Tilaveridis I., Iordanidis S., Zaraboukas T., Epivatianos A. (2010). Infiltrating angiolipoma of a the oral cavity: report of a case and literature review. *Journal of Oral and Maxillofacial Surgery*.

[13] Ali M. H., El Zuebi F. (1996). Angiolipoma of the cheek: report of a case. *Journal of Oral and Maxillofacial Surgery*.

